# Influence of Cysteine and Tryptophan Substitution on DNA-Binding Activity on Maize α-Hairpinin Antimicrobial Peptide

**DOI:** 10.3390/molecules21081062

**Published:** 2016-08-13

**Authors:** Daniel A. Sousa, William F. Porto, Maria Z. Silva, Tatiane R. da Silva, Octávio L. Franco

**Affiliations:** 1 Programa de Pós Graduação em Patologia Molecular, Universidade de Brasília, Brasília, DF 70910-900, Brazil; amarods@gmail.com; 2 Centro de Análises Proteômicas e Bioquímicas, Programa de Pós-Graduação em Ciências Genômicas e Biotecnologia, Universidade Católica de Brasília, Brasília, DF 70790-160, Brazil; Williamfp7@gmail.com (W.F.P.); marizaban@gmail.com (M.Z.S.); tatyrodrigues8@gmail.com (T.R.d.S.); 3 S-Inova Biotech, Programa de Pós-Graduação em Biotecnologia, Universidade Catolica Dom Bosco, Campo Grande, MS 79117-900, Brazil

**Keywords:** α-hairpinin, DNA-binding, antimicrobial peptides

## Abstract

For almost four decades, antimicrobial peptides have been studied, and new classes are being discovered. However, for therapeutic use of these molecules, issues related to the mechanism of action must be answered. In this work, the antimicrobial activity of the hairpinin MBP-1 was studied by the synthesis of two variants, one replacing cysteines and one tryptophan with alanine. Antibacterial activity was abolished in both variants. No membrane disturbance, even in concentrations higher than those required to inhibit the bacteria, was observed in SEM microscopy. The gel retardation assay showed that MBP-1 possesses a higher DNA-binding ability than variants. Finally, molecular modelling showed that the lack of cysteines resulted in structure destabilization and lack of tryptophan resulted in a less flexible peptide, with less solvent assessable surface area, both characteristics that could contribute to absence of activity. In summary, the data here reported add more information about the multiple mechanisms of action of α-hairpinins.

## 1. Introduction

The lack of novel tools for the control of human infectious diseases constitutes an enormous challenge nowadays. Since antibiotics are a source of evolutionary pressure on bacteria, resistance phenomena are inevitable and new antimicrobials will apparently always be necessary [1]. Based on this problem, many researchers have focused on searching for and characterizing new anti-infective molecules, including antimicrobial peptides (AMPs), which have been classified as models for innovative and promising anti-infective drugs [2].

Antimicrobial peptides are components of the innate immune system of virtually all forms of organisms, and they were maintained during the co-evolution between eukaryotes and prokaryotes [3]. They were recently described as promiscuous molecules based on their range of activities such as antiviral [4], antifungal [5], antibacterial [6], insecticidal [7] and immunomodulatory [8] activity. AMPs typically possess a single short amino acid residue chain (10–50 aa), and most of them show the presence of hydrophobic and cationic residues [9]. Furthermore, AMPs are usually classified into families based on structural fold patterns, due to the slight homology observed between primary sequences among higher groups [10]. Recently, the identification of a novel and unusual AMP motif was reported by Nolde et al. [11], based on NMR data obtained from the EcAMP-1 peptide isolated from seeds of Barnyard grass (*Echinoa crus-galli*). This peptide presents activity against several fungal phytopathogens, and its structure was characterized as two paired α-helices connected by two disulfide bridges [11,12]. Aligning the EcAMP-1 sequence with other plant peptides, a typical cysteine spacing (C^1^X_3_C^2^X_n_C^3^X_3_C^4^) was observed [11]. The name of this family, α-hairpinins, was suggested by Oparin [12], based on the similarity of the three-dimensional structure to a hairpin. Although a similar motif has been found in some animal toxins [13], the α-hairpinins were mainly characterized in plants, as observed by sequence similarity [11,12,14,15,16,17,18]. Furthermore, the helix-turn-helix motif that characterizes the α-hairpinins could be observed in snakins and thionins [19,20]. Several activities have already been reported for this family, such as trypsin inhibitors [12], antibacterial [14] and antifungal [11], indicating that like other AMPs, α-hairpinins can present multiple functions or promiscuity in plant defense [21]. The MBP-1 (maize basic peptide 1) belongs to the α-hairpinin family [14]. This peptide showed activity against *Escherichia coli*, *Clavibacter michiganensis* and the fungi from *Aspergilus*, *Sclerotinia*, *Alternaria* and *Fusarium* genera. Although MBP-1 was the first α-hairpinin discovered, the three-dimensional structure and mechanism of action were not resolved until now. In this context, our work aims to identify features in MBP-1 related to antibacterial activity. For this, variants of MBP-1 were created, replacing different sets of cysteines involved in structure stabilization with disulfide bridges, and replacing tryptophan, involved in hydrophobic interactions with bacterial membranes and DNA, with alanine. The data here reported contribute to a better understanding of the mechanisms of action of antimicrobial peptides, especially in the α-hairpinin family, against bacteria.

## 2. Results

### 2.1. Synthesis, Folding, Purification and Activity of MBP-1

In the present work, the mechanism of antibacterial activity of MBP-1 was investigated. For this, initially, MBP-1 was chemically synthesized. In order to form disulfide bonds and achieve its natural structure, 20% DMSO was used as oxidant [22]. After treatment, the loss of 4 Da observed by MS indicates that disulfide bridges were formed (Figure S1). Additionally, DMSO was removed through reversed-phase HPLC chromatography (data not shown). In order to find a model organism to investigate the activity of MBP-1, this peptide was challenged against Gram-positive (*S. aureus*) and Gram-negative bacteria (*P. aeruginosa*, *K. pneumoniae* and DH5-α and ATCC 8739 *E. coli* lineages) (Figure S2). Higher activity was observed against *E. coli* DH5-α, near to total inhibition in the loading dose of 50 µM, but no inhibition was observed in *S. aureus*.

### 2.2. Design and Activity of MBP-1 Variants

Two variants of MBP-1 were designed for this study. The first, Var 1 (W20A), was developed to study the influence of Trp at the center of the hydrophobic core of MBP-1, concerning the relationship between hydrophobicity and bactericidal activity. In the second variant (Var 2), all Cys were replaced by Ala, in order to investigate the role of disulfide bridges in MBP-1 activity. These two variants were submitted to folding by DMSO oxidation and purification. The oxidation was confirmed by MALDI analysis (Table 1), with the expected decrease of 4 Da in MBP-1 and Var 1 related to two disulfide bridge formation en each peptide. After that, MICs were determined against *E. coli* DH5-α (Table 1). No significant activity was observed in Var 1 or Var 2 until a concentration of 400 µM.

### 2.3. Scanning Electron Microscopy

In order to investigate the effects of MBP-1 and Var 1 on membranes of individual *E. coli* cells, SEM was used. At MIC levels (50 µM) and double MIC levels, the external morphology of cells treated with MBP-1 appears similar to Var 1 treated cells and the control sample, without peptide. None of the peptides seem to have disturbed the bacterial membrane (Figure S3).

### 2.4. DNA Binding-Assay

**Figure 1:**
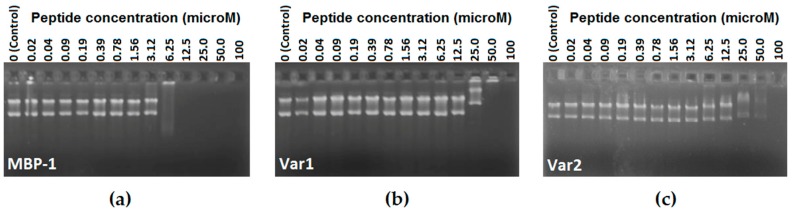
Binding activity assay of MBP-1 (**a**), Var 1 (**b**) and Var 2 (**c**) with plasmid DNA (pET28a+, 100 ng). The interaction of peptides with plasmid DNA was assessed by measuring the retardation of plasmid DNA migration through a 1% agarose gel. The peptide concentration indicated in each lane represents a serial increase in concentration from 0.02 μM to 100 μM. Plasmid DNA was only used as control in the 0 lane.

Several antimicrobial peptides can inhibit bacterial growth without cell lysis or membrane permeabilization [23]. In order to test MBP-1, Var 1 and Var 2 DNA binding ability, these peptides were evaluated in a gel retardation assay. By this method, different amounts of peptides (0.02–100 μM) were mixed with a fixed amount (100 ng) of plasmid DNA and the complexes electrophoresed on a 1% agarose gel (Figure 1). For MBP-1, until the concentration of 3.12 μM, the plasmid DNA was still able to migrate into the gel in the same way as the free DNA, while only at concentrations higher than 25 μM was the retardation of DNA observed for Var 1 and Var 2.

### 2.5. Structural Analysis

**Figure 2:**
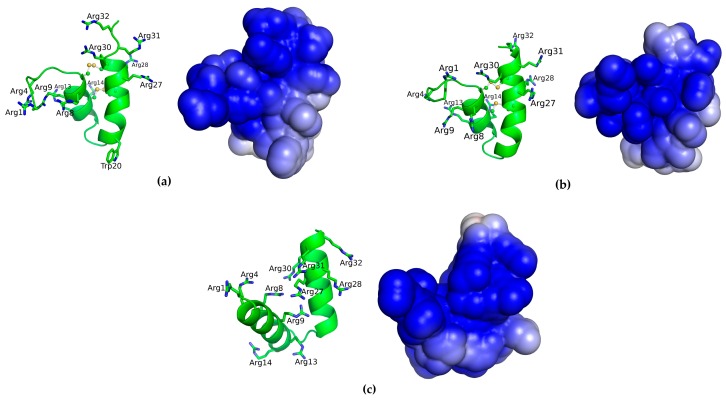
Three-dimensional models of MBP-1 and their variants. Right images represent the electrostatic surface of peptides, where blue represents positive areas and white represents uncharged areas. The models of MBP-1 (**a**); Var 1 (**b**) and Var 2 (**c**) show, respectively, a DOPE score of −1943.4, −1862.9 and −2328.6; a *Z*-Score of −4.43, −5.01 and −3.21; and 96.8%, 96.8% and 100% or residues in favored regions of Ramachandran Plot.

In order to access the structure/activity relationship between of MBP-1 and their variants, molecular modeling followed by molecular dynamics was performed. The models of MBP-1 and Var1 were constructed using the structure of EcAMP-1 (PDB ID: 2L2R) [11]. EcAMP-1 shares more than 60% of identity to MBP-1, being the most suitable template for comparative modelling. While for Var 2, an ab initio structure was generated, since the replacement of cysteine by alanine residues do not forces the peptide to adopt the native fold. Overall, the three peptides adopt a structure comprising a helix-turn-helix motif, even for Var 2 (Figure 2). In addition, the electrostatic surface of each peptide showed that most of MBP-1 and its variants’ surfaces are positively charged, which is associated with arginine residues (Figure 2), where the Var 2 has the most cationic surface, followed by MBP-1 and Var 1, according to the solvation potential energy (Table 2).

**Figure 3:**
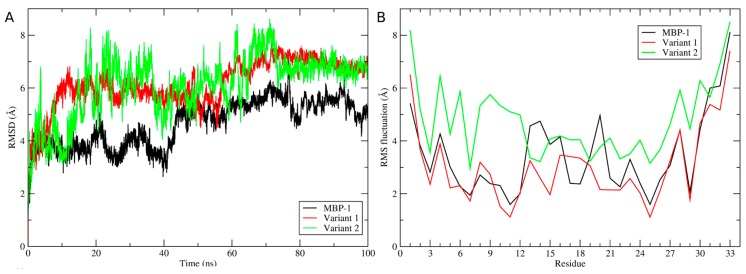

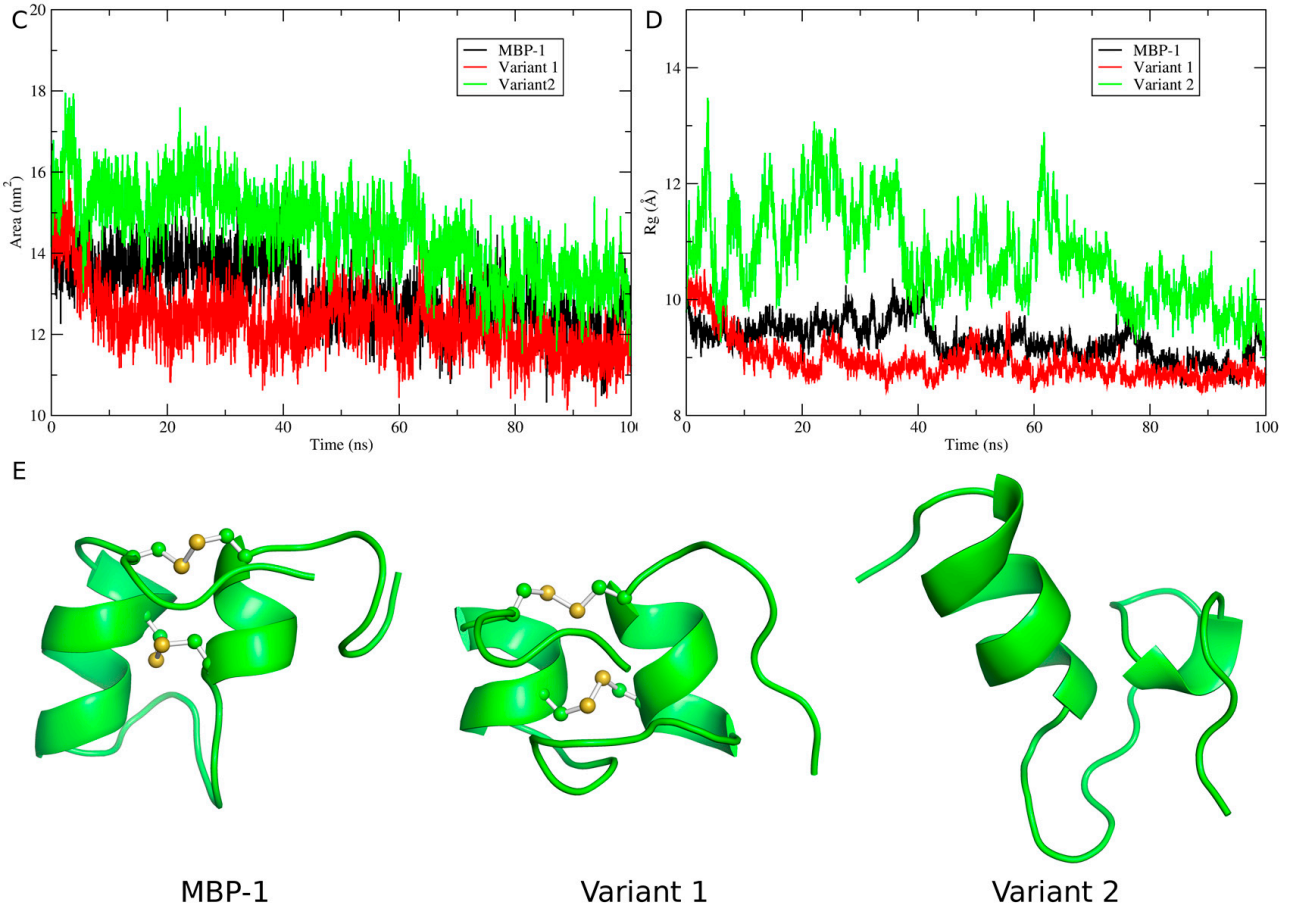
Analysis of molecular dynamics trajectories of MBP-1 (black), variant 1 (red) and variant 2 (green). (**A**) Backbone RMSD variation during the simulations; (**B**) RMS fluctuation by residue; (**C**) the solvent accessible surface area; (**D**) the variation of radius of gyration during the simulations; and (**E**) the final structures at 100 ns of simulation.

During the molecular dynamics simulations, it was observed that MBP-1 and its variants showed an RMSD variation higher than 4 Å, indicating that an unfolding process occurred (Figure 3A). Through the RMSF, it can be observed that the unfolding process of MBP-1 and variant 1 is related to the central residues ^13^RRHEGQPWETQ^23^, which showed a fluctuation of about of 3 Å (Figure 3B), while for variant 2, the process occurred in the whole structure due to the absence of disulfide bridges. In fact, the removal of disulfide bridges of MBP-1 seem to completely change its structure, in addition to the solvation potential energy (Table 2), the solvent accessible surface area (SASA) of Var 2 is higher than MBP-1 and Var 1 (Figure 3C); and the radius of gyration indicated that MBP-1 and Var 1 are more compact than Var 2 (Figure 3D). Final structures of a 100 ns simulation of MBP-1 and variants (Figure 3E) illustrates the possible structural changes caused by the amino acid modifications.

**Figure 4:**
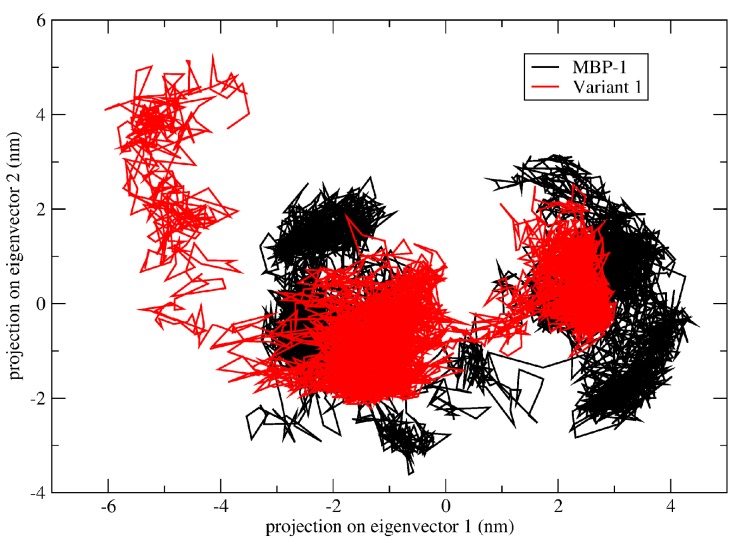
Motion projection in phase space along the first two principal eigenvectors of MBP-1 (black) and variant 1 (red). The trace of covariance matrix of MBP-1 and variant 1 was 11.287 and 9.989 nm^2^, respectively.

Once MBP-1 and Var1 showed similar SASA and radius of gyration, essential dynamics were performed in order to investigate the effect of Trp20Ala substitution. Essential dynamics have been extensively applied to study the effects of a single amino acid substitution in protein structure [24,25,26]. Through this technique, the protein motion is decomposed into several motions of different amplitudes (eigenvectors). Comparing the two motions of higher amplitude, variant 1 seems to be less flexible than MBP-1 (Figure 4), with a trace of covariance matrix of 9.989 and 11.287 nm^2^, respectively, which can also contribute to less interaction with DNA.

## 3. Discussion

In the present study, MBP-1 was chemically synthesized, folded, purified and its bactericidal potential was accessed, showing activity only against tested Gram-negative bacteria. Duvick [14] found the same activity against *E. coli* DH5-α, in addition to activity against the Gram-positive bacterium *C. michiganensis* subsp. *nebraskensis*, a *Zea mays* pathogen. Another α-hairpinin, MiAMP2d, was also tested against the bacteria *C. michiganensis*, *Ralstonia solanacearum* and *E. coli* [15], but only presented activity against *E. coli*. SmAMP3, an α-hairpinin isolated from *Stellaria media*, was challenged against several bacteria and fungi, but only presented antifungal activity [27]. It is possible that other members of the α-hairpinin family have antibacterial activity, but only SmAMP3, MiAMP2d and MBP-1 have been challenged against bacteria until now in the literature.

To obtain a better understanding of the molecular mechanism of MBP-1 antimicrobial activity, two variants were also synthesized. It was hypothesized that the lack of cysteines, compromising structure, could affect antimicrobial activity in Var 2. Also, replacement of Trp by Ala in our study (Var 1) would have great impact on the activity due to diminution of the hydrophobic surface, responsible for membrane mediated interactions. Antibacterial activity was abolished in both variants, but no membrane disturbance in *E. coli* cells was observed in SEM to MBP-1 or Var 1, indicating an intracellular mechanism of action.

Despite the classic action mechanism of AMPs involving their ability to cause cell membrane damage, much evidence indicates that some AMPs can interact with intracellular targets inducing cell damage. Some AMPs have been reported as having inhibitory activity due to impairment of DNA translation by direct binding. Studies with puroindolines, AMPs isolated in wheat, suggests that AMPs with high net positive charge, such as MBP-1, are associated with DNA binding activity [28]. DNA retardation assay showed that MBP-1 has a higher DNA affinity than variants, which indicates that the tryptophan and cysteines might be relevant to its DNA-binding mediated antimicrobial activity. Other AMPs also have DNA-binding activity reported, such as buforin II, which is able to cross the cell membrane without permeabilizing it, later accumulating in the cytoplasm and binding to DNA and RNA [29]. Indolicidin, one of the smallest natural cationic peptides, may also act in inhibiting DNA synthesis [30], as may cathelicidin PR-39 [31].

A previous report suggests that peptides with the most potent antibacterial activities have the greatest affinity for the plasmid DNA [32]. The agarose gel retardation assay suggests, as indirect evidence, that MBP-1 can interact with transcription or translation processes in bacteria. Studies with the AMPs puroindolines suggests that peptides with the most potent antibacterial activities have the greatest affinity for the plasmid DNA, also inhibiting the transcription and translation process in vivo [32]. Furthermore, the AMPs cathelicidins also have affinity for plasmid DNA. This peptide crosses the cell, without causing membrane damage, and blocks DNA and protein synthesis in bacteria, inhibiting growth [31].

Molecular modeling was used to better understand the difference in activity of MBP-1 and variants. Through these studies, it could be observed that arginine residues confer to MBP-1 a large positive surface area. However, the DNA-binding property is not only related to electrostatic interactions between the positively charged peptide and the polyanionic DNA. The electrostatic interactions between peptides and DNA seem to be directed related to isoelectrical point [33]. In our observations, MBP-1 and Var 1 possesses the same isoeletrical point (11.35) and Var 2 a slightly higher (12.08). Thus, their DNA binding activity should be greater or equal to the original peptide, showing that other features are possible responsible for DNA interaction.

Structural analysis demonstrates that removing the disulfide bridges of MBP-1 generates drastic changes, which could be involved in a loss of activity due to diminished DNA affinity. For hepicidin, a β-hairpinin antimicrobial peptide, replacement of cysteines with alanine residues led to peptides that were no longer able to bind DNA [34]. Other peptides like murine β-defensin Defr1 [35], human neutrophil defensins HNP-1 [36], human β-defensin 2 [37], Medicago NCR antimicrobial peptides [38] and tachyplesin also showed reduced antimicrobial activity after linearization [39]. Slightly higher accessible surface area could explain MBP-1’s greater DNA affinity than Var 1. Although variant 2 has a higher solvent accessible surface area, which could be a result of separation of the α-helices, lack of three-dimensional structure may be responsible for a lower activity.

In the present study, molecular models are theoretical, but they are reliable, since models constructed for MBP-1 and Var 1 share more than 60% of identity with the template used. The same standard applies to Var 2, modeled by ab initio techniques. The QUARK server has been ranked among the best prediction servers in CASP and it is accurate for predicting structures with helix-turn-helix motifs, as observed for snakin-1 structure, which was firstly predicted by QUARK [20] and some years later the helix-turn-helix was confirmed by crystallography [19].

## 4. Materials and Methods

### 4.1. Synthesis, Purification and Folding of MBP-1 and Variants

Firstly, the original sequence of MBP-1, together with two variants were designed (Table 1). Lyophilized peptides were purchased from China Peptides Co. Ltd. (Shanghai, China) and then re-suspended in 500 µL of ultrapure water. For further disulfide bridges formation and folding, 0.5 mg of peptide was mixed with 10 mL of 20% DMSO, pH 8.7, for 7 h at room temperature. In order to remove DMSO, peptides were diluted in 0.2% TFA (1:1) and applied onto a reversed-phase HPLC column (Vydac C18 TP522, Grace, Columbia, MD, USA) and eluted with a linear acetonitrile gradient (5%–50%-15 min), at flow rate of 1 mL·min^−1^. Peptide elution was monitored at 216 nm. Collected fractions were also checked by mass spectrometry (MALDI-TOF/TOF Ultraflex II—Bruker Daltonics, Bremen, Germany).

### 4.2. Antibacterial Bioassays

*Escherichia coli* DH5-α, *E. coli* ATCC 8739, *Staphylococcus aureus* ATCC 29213, *Pseudomonas aeruginosa* ATCC 27853, *Klebsiella pneumoniae* ATCC 13882 were used to evaluate antibacterial activity. Bacterial cells were previously replicated in liquid Mueller-Hinton (MH) medium, under stirring for 2–3 h, at 37 °C. Antimicrobial activities against five bacterial lineages were determined using the broth micro-dilution method described previously by CLSI (2012). The tests were conducted in multi-well plates from the original culture. For this, inoculum was prepared, first being diluted to approximately 5 × 10^4^ UFC·mL^−1^ per well. In order to determine the minimum inhibitory concentration (MIC) of MBP-1, further variants were realized, using a peptide serial dilution starting from 400 µM to 3 µM. The peptide solution volume did not exceed 10% of well volume of the containing liquid MH. Multi-well plates (TPP—96 well) were inoculated and then incubated with slight agitation at 37 °C for 2–3 h. Bacterial growth was monitored at 595 nm every 30 min during the exponential phase. The MIC was determined as the lowest concentration that inhibits 100% of bacterial growth. The amount of bacterial growth was determined comparing the growth of the positive control with negative control. Sterile distilled water was used as negative control and chloramphenicol (25 mM) was used as a positive control in all experiments.

### 4.3. Scanning Electron Microscopy

*E. coli DH5*-α cultures were grown overnight in 10 mL of LB. Aliquots of 200 μL of this bacterial suspension were incubated with 800 μL of MBP-1 or Var 1 at final concentration of 50 and 100 μM in fresh LB, for 2.5 h at 37 °C. The cells were collected by centrifugation (10,000 *g*, 5 min), and the pellets were washed three times in PBS (pH 7.4) and re-suspended in 100 mL MilliQ (ultra-pure) water. An aliquot of the cell suspension was spotted onto a glass slide, air-dried, then fixed and dehydrated. For this, the air-dried slides were fixed in 2.5% glutaraldehyde (in PBS, pH 7.4) overnight in a humid chamber, then washed with PBS pH 7.4 for 10 min, and dehydrated in an ethanol gradient of 50%, 60%, 70%, 80%, 90% and 100%. The slides were coated in a Dynavac CS300 coating unit with gold, with double-sided conducting carbon tape attached to the slides for better conductivity. The samples were analyzed using a ZEISS supra 10 VP field emission scanning electron microscope (Carl Zeiss Microscopy, Thornwood, NY, USA) at 3.0 kV.

### 4.4. DNA Binding-Assay

Gel retardation experiments were performed as described [28] with slight modifications. For this, 100 ng of the plasmid DNA (pET 28a+) were mixed with a serial dilution of each peptide (100 μM to 0.2 μM) in 20 μL of ultrapure water and incubated at room temperature for 1 h. Subsequently, 2.5 μL of modified loading buffer (10 mM Tris-HCl, pH 7.5, glycerol, 50 mM EDTA, 0.25% bromophenol blue and 0.25% xylene cyanol) was added, and electrophoresed in 1% agarose gel. After that, the gels were immersed in EtBr solution (0.5 μg·mL^−1^) for 30 min before imaging.

### 4.5. Comparative Molecular Modelling

The HHPred server [40] was used for MBP-1 template identification. Then, five hundred molecular models of MBP-1 and Var 1 were constructed by comparative molecular modeling through MODELLER 9.10 [41] using the structure of EcAMP-1 (PDB ID: 2L2R) [11]. The models were constructed using the default methods of automodel and environ classes from MODELLER. The final models were selected according to the discrete optimized protein energy score (DOPE score). This score assesses the energy of the model and indicates the best probable structures. Ab initio modelling was applied for Var 2, by means of the QUARK server [42] and the model was also evaluated DOPE score, using MODELLER. The best models were evaluated through PROSA II [43] and RAMPAGE [44]. RAMPAGE checks the stereochemical quality of a protein structure through the Ramachandran plot, where good quality models are expected to have more than 90% of amino acid residues in most favored and additional allowed regions, while PROSA II indicates the fold quality. The electrostatic surface was calculated by means of APBS [45]; firstly, the conversion of pdb files into pqr files was perfomed by the utility PDB2PQR using the AMBER force field [46]. The grid dimensions for APBS calculation were also determined by PDB2PQR. Solvation potential energy was calculated by APBS. Structure and surface visualization was done in PyMOL (http://www.pymol.org).

### 4.6. Molecular Dynamics Simulation and Analyses of Molecular Dynamics Trajectories

The molecular dynamics simulations of the ensembles (MBP-1 and variants) were carried out in water environment, using the Single Point Charge water model [47]. The analyses were performed by using the GROMOS96 43A1 force field and computational package GROMACS 4 [48]. The dynamics used the MBP-1 and variants three-dimensional models as initial structures, immersed in water, in cubic boxes with a minimum distance of 1 nm between the peptides and the edges of the boxes. Chlorine ions were also inserted into the complexes with positive charges in order to neutralize the system charge. Geometry of water molecules was constrained by using the SETTLE algorithm [49]. All atom bond lengths were linked by using the LINCS algorithm [50]. Electrostatic corrections were made by Particle Mesh Ewald algorithm [51], with a cut-off radius of 1.4 nm in order to minimize the computational time. The same cut-off radius was also used for van der Waals interactions. The list of neighbors of each atom was updated every 20 simulation steps of 2 fs. The system underwent an energy minimization using 50,000 steps of the steepest descent algorithm. After that, the system temperature was normalized to 300 K for 100 ps, using the velocity rescaling thermostat (NVT ensemble). Then the system pressure was normalized to 1 bar for 100 ps, using the Parrinello-Rahman barostat (NPT ensemble). The systems with minimized energy, balanced temperature and pressure were simulated for 50 ns by using the leap-frog algorithm. Molecular dynamics simulations were analyzed by means of the backbone root mean square deviation (RMSD), residue root mean square fluctuation (RMSF), radius of gyration and solvent accessible surface area using the g_rms, g_rmsf, g_gyrate and g_sas built-in functions of the GROMACS package [48], respectively. The essential dynamics was used to analyze and visualize the overall motions of simulation. The covariance matrices of wild type and variant peptides were constructed using the main chain atoms. The essential dynamics was performed using the g_covar and g_anaeig utilities of the GROMACS package. Three snapshots were taken from the trajectories in 30, 40 and 50 ns for solvation potential energy calculation. The snapshots were taken using the trjconv utility from GROMACS.

## 5. Conclusions

In the present study, we report the first evidence for the DNA binding mechanism of MBP-1, an antibacterial peptide from the α-hairpinin family. These results contribute to a better understanding of the mechanism of action of this antimicrobial peptide family, and could be used for the design of potent antimicrobial peptides with therapeutic application.

## Figures and Tables

**Table 1:** Sequences, masses before and after oxidation and MIC of MBP-1 and its variants. Mutated residues are underlined in the sequence. Mass/charge relation before and after oxidation was checked by MALDI.

Peptide	Sequence	Mass before Oxidation	Mass after Oxidation	MIC against *E. coli*
MBP-1	RSGRGE**C**RRQ**C**LRRHEGQP**W**ETQE**C**MRR**C**RRRG	4125.69 *m*/*z*	4121.04 *m*/*z*	50 μM
Var 1	RSGRGECRRQCLRRHEGQP**A**ETQECMRRCRRRG	4008.05 *m*/*z*	4004.01 *m*/*z*	>400 μM
Var 2	RSGRGE**A**RRQ**A**LRRHEGQPWETQE**A**MRR**A**RRRG	4001.40 *m*/*z*	4001.38 *m*/*z*	>400 μM

**Table 2:** Solvation potential of MBP-1 and variants 1 and 2.

Peptide	Solvation Potential Energy (KJ/mol)
MBP-1	3399.486
Var 1	3373.256
Var 2	3487.306

## Supplementary Materials

The following are available online at http://www.mdpi.com/1420-3049/21/8/1062/s1, Figure S1: MBP-1 mass spectrum before (higher peak—4125.69) and after folding (lower peak—4121.04), showing 4 kDa decrease related to cysteine oxidation, Figure S2: Bactericidal assays. Five bacterial lineages were incubated with 50 µM of MBP-1. After 2.5 h it was possible to observe the difference in inhibition, which was more pronounced in *E. coli* DH5-α. This strain was chosen for subsequent assays. Chloramphenicol (0.25 mM) was used as positive control, and ultrapure water as negative control, Figure S3: Scanning electron microscopy of *E. coli* cells, treated with MBP-1 at 50 μM (A); 100 μM (B) and Var 1 at 50 μM (C); 100 μM (D); No peptide as control (E). Scale bar 1 μm. Magnification 14,000× (A,B,D), 13,000× (C) and 15,000× (E).
