# Supplementary material for: Influence of Cysteine and Tryptophan Substitution on DNA-Binding Activity on Maize α-Hairpinin Antimicrobial Peptide
Source: Molecules. 2016 Aug 13;21(8):1062. doi: 10.3390/molecules21081062 (PMC6273665; doi:10.3390/molecules21081062)
Supplement: Supplementary file 1 [file molecules-21-01062-s001.pdf]

Supplementary Materials: Influence of Cysteine and Tryptophan Substitution on DNA-Binding Activity on Maize Alpha-Hairpinin Antimicrobial Peptide

Daniel A. Sousa, William F. Porto, Maria Z. Silva, Tatiane R. da Silva and Octávio L. Franco

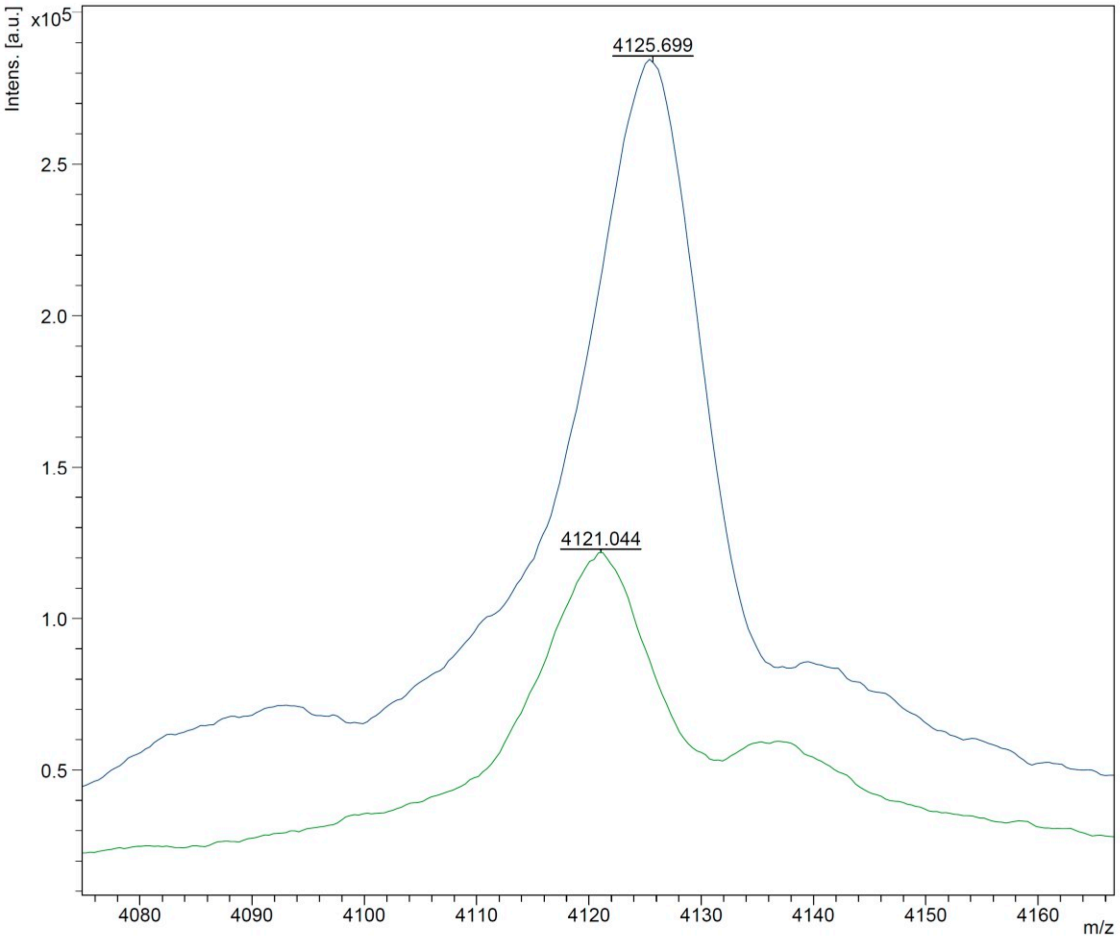

Figure S1. MBP-1 mass spectrum before (higher peak—4125.69) and after folding (lower peak—4121.04), showing 4 kDa decrease related to cysteine oxidation.

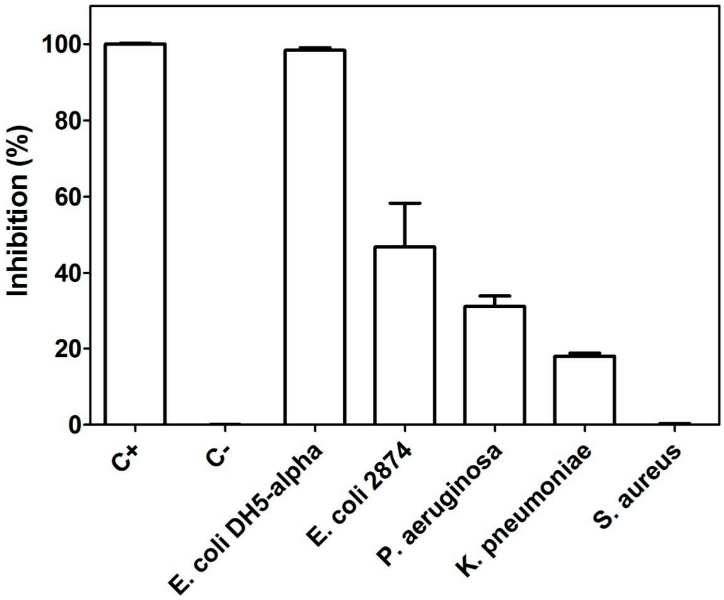

Figure S2. Bactericidal assays. Five bacterial lineages were incubated with 50 µM of MBP-1. After 2.5 h it was possible to observe the difference in inhibition, which was more pronounced in *E. coli* DH5-*α*. This strain was chosen for subsequent assays. Chloramphenicol (0.25 mM) was used as positive control, and ultrapure water as negative control.

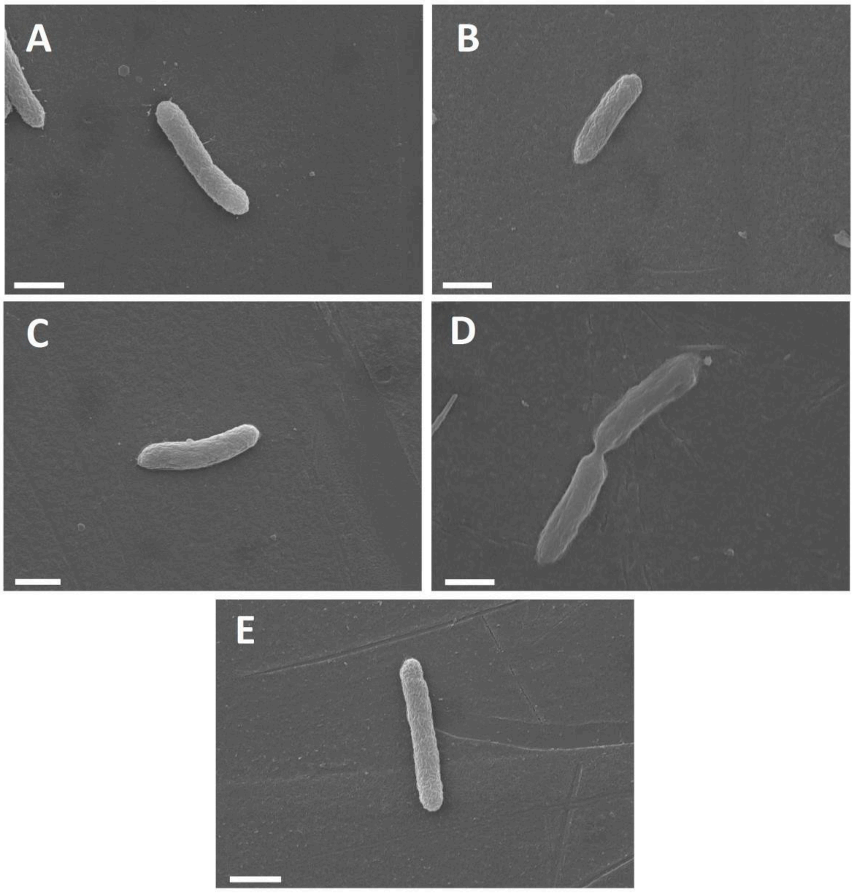

Figure S3. Scanning electron microscopy of *E. coli* cells, treated with MBP-1 at 50 µM (**A**); 100 µM (**B**) and Var 1 at 50 µM (**C**); 100 µM (**D**); No peptide as control (**E**). Scale bar 1 µm. Magnification 14,000× (**A**,**B**,**D**), 13,000× (**C**) and 15,000× (**E**).
